# The Association of Nil Per Os (NPO) Days with Necrotizing Enterocolitis

**DOI:** 10.1155/2018/2795468

**Published:** 2018-10-30

**Authors:** Yongming Wang, Xiaoyu Li, Chunbao Guo

**Affiliations:** ^1^Ministry of Education Key Laboratory of Child Development and Disorders, Children's Hospital, Chongqing Medical University, Chongqing, China; ^2^Department of Neonatology, Children's Hospital, Chongqing Medical University, Chongqing, China; ^3^Department of Pediatric General Surgery and Liver Transplantation, Children's Hospital, Chongqing Medical University, Chongqing, China

## Abstract

**Background:**

Enteral feeds are an essential part of care for infants and may be a potential risk factor in NEC development. The present study objective was to evaluate the relationship between nil per os (NPO) and clinical outcomes in infants with NEC.

**Methods:**

This was a retrospective review of 196 premature, low-birth-weight infants with NEC from January 1, 2011, to October 31, 2016, at four academic tertiary care hospitals. The patients were evaluated based on the median nil per os (NPO) days (5.6 days) in longer NPO (6.3 ± 1.1 days) versus shorter NPO groups (4.2 ± 0.9 days).

**Results:**

Patients who experienced longer than 5.6 NPO days were more likely associated with perforated NEC (odds ratio (OR), 2.01; 95% confidence interval (CI), 1.07–3.76; *p* = 0.021), stage III NEC (OR, 1.81; 95% CI, 0.97–3.38; *p* = 0.042), and longer duration of mechanical ventilation (OR, 0.17; 95% CI, 0.08–0.98; *p* = 0.005) than the shorter duration group of 5.6 NPO days. For the secondary outcomes, there was a trend towards earlier birth (*p* = 0.083), longer NICU length of stay (*p* = 0.093), and higher mortality (*p* = 0.10) in the longer NPO cohort (*p* = 0.057). The incidence of bacterial sepsis and short bowel syndrome also increased as the length of NPO increased. There was no statistically significant difference in nutritional variables between the two groups within the in-hospital period.

**Conclusion:**

Longer NPO time was associated with the severity of NEC and more injurious clinical outcomes, as demonstrated by rates of surgical intervention and duration of mechanical ventilation.

## 1. Introduction

Necrotizing enterocolitis (NEC) is a major gastrointestinal emergency in neonates that affects approximately 6 to 7% of very-low-birth-weight (VLBW) infants born weighing less than 1500 g [1–3]. Numerous factors are thought to be associated with the pathogenesis of NEC, including prematurity, infection, red blood cell transfusion, enteral feeding, and bacterial colonization, which often lead to an inflammatory state and the clinical manifestation of NEC [4–6]. Enteral feeding of the premature gut, which has served as a therapeutic and preventive strategy in the management of NEC, has been implicated in both the pathogenesis and development of NEC [7, 8]. The current clinical practice includes a widely varied, cautious, delayed approaches to feeding very-low-birth-weight (VLBW) infants for fear of NEC, which may cause postnatal growth restriction.

It is thought that the proteins and carbohydrates derived from enteral nutrition provide substrates for bacterial growth and fermentation, which are possible risk factors for NEC. Conversely, enteral nutrition can prevent microbial overgrowth and prevent inflammation in the preterm gut [9]. The common thought is that early human milk enteral feeding appears to be a prevention strategy to reduce the occurrence of NEC. In contrast, withholding feedings and enforcing nil per os (NPO) in infants may have their own unintended consequences, including duodenal mucosal atrophy, impaired intestinal function, abnormal gut permeability, and subsequent feeding intolerance [10]. Furthermore, only parenteral feeding has been associated with various complications, including central line infections, liver disease, and alteration of the intestinal flora in the premature gut [11]. Several studies have shown that enteral nutrition has trophic effects that can minimize some of the intestinal problems caused by total parenteral nutrition [12, 13]. The potential for increasing the risk for NEC by delaying or interrupting enteral nutrition lies in the aforementioned benefits of enteral nutrition as it impacts gastrointestinal immune function, maturation, and integrity.

Currently, there are few published studies addressing whether or not there is any relationship between delayed or interrupted enteral nutrition and the development of NEC in extremely preterm neonates. We hypothesized that a longer duration of NPO (NPO days) would increase NEC severity in premature, low-birth-weight infants. We therefore conducted the present study to address the association between the number and percentage of nil per os (NPO) days and NEC in extremely preterm neonates, especially in the context of developing countries.

## 2. Materials and Methods

### 2.1. Patient Cohort

A retrospective review of the hospital-based cohort for NEC from January 1, 2011, to October 31, 2016, was performed in 4 newborn intensive care units (NICUs) at Yongchuan Hospital, Children's Hospital of Chongqing Medical University, Sanxia Hospital, and Jinan Maternity and Child Care Hospital with the approval of the institutional review board at each site. Infants eligible for inclusion had a diagnosis of NEC and Bell's stage ≥ stage 2 during the study period if they were premature (<37-week gestational age) at birth and had a very low birth weight (≤1500 g). Exclusion criteria included infants conceived from in vitro fertilization (IVF), acute pulmonary bacterial infection, major congenital anomalies (congenital heart disease), and gastrointestinal abnormalities (imperforate anus, intestinal atresia, or Hirschsprung disease), or infants without the information needed for outcome measures. All infants with NEC were followed up in the hospital's outpatient clinic until at least 3 months of corrected age. The diagnosis of NEC was defined using Bell's criteria and modified by Walsh and Kliegman [13], including clinical findings of abdominal distension, feeding intolerance, and radiographic findings (the presence of pneumatosis intestinalis, pneumoperitoneum, and/or portal gas). The SNAP-II (Score for Neonatal Acute Physiology, version II) was calculated from the data from the 1st day of life. The data pertaining to NEC that had been recorded by physicians or nurses during patient hospitalization were collected from the electronic medical records of patients, including demographic information, diagnosis, clinical management, laboratory data, mortality, length of stay (LOS), and discharge summaries.

### 2.2. Clinical Management and Follow-Up

All the patients were subjected to the same medical program, including feeding regimen, fluid resuscitation, parenteral nutrition support, and perioperative broad-spectrum antibiotics, if necessary. Here, the feeding regimen was directed by the discretion of the clinicians. Breast milk and human milk fortifier was primarily administered for enteral nutrition and initiated on the first day with 20 mL/kg/day and advanced by 30 mL/kg/day until maximum enteral feedings of 170 mL/kg/day were attained. If mother's milk was unavailable, a 20 cal/oz premature formula could be substituted for breast milk. All feeds were given as bolus by nasogastric tube at 2-hour intervals. Data on the type of milk or volume of milk were not collected. The feeds were discontinued for feeding intolerance and mechanical ventilation. NPO days were defined as days when an infant received no enteral nutrition over a 24-hour period. The number of NPO days from the day of life to the development of NEC was calculated. The primary outcome measure was the proportion of patients with perforated NEC and Bell's staging III. Surgical NEC was defined as NEC requiring either drainage or exploratory laparotomy. The secondary outcomes included in-hospital mortality within 90 days, total parenteral nutrition for 90 days, and the length of hospital stay for patients surviving 90 days.

### 2.3. Statistical Analysis

Statistical analyses were performed using the SPSS statistical package version 22 (SPSS Inc., Chicago, IL). Student's *t*-test and Mann-Whitney *U* test were used for comparison of parametric variables, which are described as the means ± standard deviation (the mean ± SD of the variable), and nonparametric continuous variables, which are described as medians and interquartile ranges (IQRs), respectively. Chi-square or Fisher's exact tests were used to compare categorical variables. The relative risks for postoperative variables were assessed using cross-tabulation (odds ratio (OR)) or multivariate logistic regression analysis (risk ratio (RR)). The conditional logistic regression model was used to investigate the association of the NPO day with the clinical outcome of NEC. We adjusted for the presence of a PDA, inotrope use (yes/no), antibiotic use (yes/no), and SNAP-II scores in the model (based on *p* < 0.1 in univariate analyses). The statistical significance was evaluated using a two-tailed 95% confidence interval (CI), and a *P* value less than 0.05 was considered statistically significant.

## 3. Results

### 3.1. Demographic Data

There were a total of 243 preterm infants followed for NEC diagnosis managed at our institution eligible for analysis during the study period, and 47 cases were excluded due to incomplete information (*n* = 29) and intestinal malformation (*n* = 18). The demographic characteristics of the infants are shown in Table 1. Sixty-five percent of the infants (*n* = 128) were male, and the average gestational age (GA) and birth weight of the patients were 26 ± 3.8 weeks and 1013 ± 306 g, respectively. Twenty-five percent of the infants (48, 24.5%) were assessed with over stage 3 disease by Bell's staging (Table 1), 58 (29.6%) of the included infants underwent surgical intervention after a median time period of 9 days (range: 2–19), and the remaining 138 infants were managed medically. Enteral feeding prior to NEC onset was identified in more than 77.6% (*n* = 152) of the included infants. Forty-six infants (23.5%) needed assisted ventilation, and 45 infants (23.0%) needed hemodynamic support by fluid expansion with the administration of vasoactive amines. The median age at diagnosis was 13 days, and there were 69 cases (35.2%) with probiotic administration.

To explore clinical outcomes associated with NPO days, the corrected NPO days were dichotomized as “longer” or “shorter” based on the median amount of NPO days (5.6 days, Table 1). In the longer NPO group, an average of 6.3 ± 1.2 NPO days occurred before developing NEC compared with 4.2 ± 0.9 days to development of NEC in the shorter NPO group (Table 2). The baseline characteristics and clinical status of the two groups are reported in Table 2. No differences were observed for some of the baseline characteristics, including sex (*p* = 0.52), APGAR scores (at 5 minutes) (*p* = 0.36), probiotic usage (*p* = 0.29), or other disease severity in perforated necrotizing enterocolitis. Patients in the longer NPO group were more likely to be younger at birth (*p* = 0.083) and had a lower birth weight (*p* = 0.101) than were those with low NPO days, although no statistical significance was observed. With respect to some maternal features, such as maternal age (*p* = 0.24) or delivery mode (cesarean or vaginal) (*p* = 0.44), there was no difference among the two groups, which suggested that the study population reasonably represented the spectrum of neonates with necrotizing enterocolitis in our institutions. No differences in other clinical status, such as basic laboratory measures or vasopressor use at onset of NEC (*p* = 0.38), were found between the two groups.

### 3.2. Outcomes of Patients according to NPO Days

According to the established criteria, a comparison of the outcomes between the two groups is summarized in Table 3. Patients who experienced longer than 5.6 NPO days were more likely associated with perforated NEC (odds ratio (OR), 2.01; 95% confidence interval (CI), 1.07–3.76; *p* = 0.021) and stage III NEC (OR, 1.81; 95% CI, 0.97–3.38; *p* = 0.042). Furthermore, neonates in the longer NPO group required a significantly longer duration of mechanical ventilation (OR, 0.17; 95% CI, 0.08–0.98. *p* = 0.005) and had a higher rate of death prior to discharge than did those with fewer NPO days, although no significant differences were found (*p* = 0.10) (Table 3). The cause of death was primarily attributed to pan-intestinal NEC in 29 infants. The remaining deaths resulted from sepsis (*n* = 11) and severe short bowel (*n* = 8). There was no death that occurred after stoma closure. There were no significant differences in the rates of dependence on parenteral nutrition 90 days after surgery for the 76 patients surviving at least 90 days postoperatively (*p* = 0.30).

No significant differences were found in nutritional variables between the two groups within the in-hospital period (albumin and prealbumin). A higher proportion of neonates with more NPO days were diagnosed with culture-proven sepsis (*p* = 0.16) and short bowel syndrome (*p* = 0.17) than the proportions of those patients with fewer NPO days, but there were no statistically significant differences (Table 3). The majority of sepsis (65%) in the case population occurred within 72 hours or concurrent with the diagnosis of NEC. The mean hospitalization time in the NICU (*p* = 0.093) was longer in the group with longer NPO days, but this difference was not significant.

## 4. Discussion

In this study, we identified a statistically significant association between the length of NPO days and the severity and mortality of NEC in premature infants. In this study, the length of NPO time before the development of NEC was dichotomized to facilitate comparison between NEC patients in premature, low-birth-weight infants. In our research, patients who experienced more NPO days developed NEC at an earlier age and had higher rates of perforation. Due to the higher incidence of perforated NEC in those experiencing more NPO days, there was also a higher need for surgery or longer hospital stay.

To the best of our knowledge, few studies have directly investigated the impact of delayed or interrupted enteral intake on NEC onset or severity [14]. In our study, we tried to address this question and found that patients who were on a longer NPO restriction developed NEC at an earlier age and had a higher rate of perforation than those who had fewer NPO days. Although no studies have looked directly at the impact of NPO on NEC severity, a few have examined the effects of delayed enteral feeding on NEC onset. A recent study found that more NPO days were associated with a higher rate of NEC [15]. Our study was specifically aimed at exploring the potential negative consequences of keeping infants NPO, suggesting that the length of time spent with NPO can be a risk factor for NEC severity. Of course, this finding should be taken in the context of the other known risk factors for NEC in preterm infants. Feeds were discontinued due to feeding intolerance and mechanical ventilation. Since feeding intolerance and mechanical ventilation may be signs of NEC, NEC in itself may lead to longer fasting times, which may confound the results. We cannot comment on the indication for NPO status or whether the NPO days were primarily during the first few days of life and due to a delayed initiation of feeds. Enteral nutrition might promote gastrointestinal colonization with symbiotic organisms that form the intestinal microbiome, decrease bacterial translocation, improve intestinal motility, and prevent microbial overgrowth, which are the preventative pathophysiologic mechanisms for NEC [16–18]. Inhibition of inflammatory pathways that promote the development of NEC is emerging as one example of microbiome-mediated protection of the gastrointestinal tract.

Other factors, such as infection, hypoxia, ischemia, formula milk [19], and prematurity, per se, have been considered important in the pathogenesis of NEC. Compared with the group with shorter NPO days, infants who experienced longer NPO time had higher incidences of PDA, inotrope use, and antibiotic use and higher SNAP-II scores. All these measures are indicative of confounding as these may be related to delayed introduction or interruption of feeds and are themselves associated with development of NEC. Therefore, certain authors argue that the risk of NEC should not be considered in isolation of other potential clinical outcomes while formulating feeding policies and practices for preterm infants [20]. Clinical studies have evaluated the effects of different feeding regimens on the incidence of NEC and outcomes in premature neonates [21]. The use of breast milk initially may be protective against NEC. We did use maternal breast milk or donor breast milk exclusively until the infant reached 1500 g and was on full feeding volume, then we introduced formula. This was due to the high cost of donor breast milk to our hospital. The reduction in the use of TPN was significant, and any reduction in exposure to TPN further reduces the risk for infections, as well as TPN-associated liver disease [22]. The fluids from supplemental TPN during NPO days likely aid in meeting the fluid requirements at the onset of NEC, ensuring that infants are still being sustained with parenteral fluids prior to the clinical onset of symptoms. The recent Cochrane reviews did not reveal a statistically significant effect of early enteral feeding practices on the risk of NEC or all-cause mortality in very preterm or VLBW infants. Moreover, approximately 80% of the infants in those reviews were growth restricted and had evidence of fetal circulatory distribution abnormalities [23]. However, we did observe a detrimental effect of TPN on the risk of NEC.

Inherent limitations of our study include its retrospective nature, where data regarding the NPO days was unavailable for certain patients, creating an inherent risk of selection bias. Second, there were a number of infants for whom life support was withdrawn, making it difficult to interpret the survival data. A majority of the babies were on mixed human milk and formula feeds. At discharge, however, a majority had made a successful transition to exclusive human milk feedings. Third, although the study shows benefits from feeding for preterm infants in the current cohort, the results should be interpreted with care, given the lack of long-term data to assess the impact of NPO days for overall physiological advancement. Finally, the median difference in the time to regain birth weight must be interpreted with caution, given the nonblinded design and absence of data regarding feeding practice postdischarge. The strength of our study is that it evaluates a fairly uniform patient population that was managed similarly at our institution. A larger, multi-institutional study may help better define the impact of NPO days on outcomes.

## 5. Conclusion

In the current study, we identified that neonates who spent more time NPO had an increased severity of disease, as evidenced by higher rates of perforated NEC. We speculate that delayed or interrupted provision of enteral intake to preterm neonates affects gut maturation and defenses and predisposes infants to the development of NEC in this already at-risk population. It will be necessary to conduct further prospective cohort studies to provide information about this association and the potential reasons for the development of NEC.

## Figures and Tables

**Table 1:** Baseline demographics and clinical characteristics for the eligible cohort (*n* = 196).

Variables	
Male : female	128 : 68
Gestational age (wk)	26.7 ± 3.8
Birth weight (g)	1013 ± 306
NPO (days)	5.6 ± 1.6
Bell's stage ≥ stage 3	48 (24.5)
History of enteral feeding, *n* (%)	152 (77.6)
Respiratory support, *n* (%)	46 (23.5)
Probiotics, *n* (%)	36 (12.8)
Age at diagnosis of NEC (days)	21 (5–35)
APGAR scores at 5 minutes	8.1 ± 1.3
SNAP-II scores	18.8 ± 12.6
Vasopressor use at enrollment, *n* (%)	59 (30.1)

**Table 2:** Baseline demographics of eligible patients based on NPO days (chi-square test and Student's *t*-test).

Characteristics	Longer NPO (*n* = 98)	Shorter NPO (*n* = 98)	*p* values
Male : female	65 : 34	63 : 34	0.52
Gestational age (wk)	25.8 ± 2.9	27.4 ± 3.1	0.083
Birth weight (g)	964 ± 296	1125 ± 314	0.101
Probiotics, *n* (%)	16 (16.3)	20 (20.4)	0.29
APGAR scores at 5 minutes	8.1 ± 1.2	8.2 ± 1.1	0.36
SNAP-II scores	17.9 ± 12.3	19.6 ± 11.9	0.15
Cesarean delivery, *n* (%)	65 (66.3)	67 (68.4)	0.44
Maternal age (years)	29.6 ± 5.8	30.3 ± 4.7	0.24
SGA infant, *n* (%)	11 (11.2)	12 (12.2)	0.35
Transfusion preoperation, *n* (%)	17 (17.3)	13 (13.3)	0.28
Vasopressor use at enrollment, *n* (%)	28 (28.6)	31 (31.6)	0.38
Respiratory support, *n* (%)	24 (24.5)	22 (22.4)	0.43
First platelet count (onset of symptoms) (10^9^/L)	288.2 ± 95.2	297.6 ± 88.6	0.16
Hemoglobin (g/L)	136.2 ± 41.3	129.8 ± 39.5	0.32
First WBC (onset of symptoms) (10^9^/L)	17.8 ± 3.7	17.2 ± 4.8	0.42
First procalcitonin (onset of symptoms) (ng/mL, normal value: 0–0.5)	5.9 ± 3.4	6.3 ± 3.2	0.14
First CRP (onset of symptoms) (mg/L, normal value: 0–10)	25.3 ± 9.7	24.8 ± 8.9	0.18
Albumin (g/L, normal range, 35–50)	29.2 ± 6.2	28.9 ± 5.8	0.12

**Table 3:** Patient outcomes based on the NPO days.

	Longer NPO (*n* = 98)	Shorter NPO (*n* = 98)	*p* values	Odds ratio (95% CI)
Perforated NEC	36 (36.7)	22 (22.4)	0.021	2.01 (1.07–3.76)
NEC stage				
Stage II	63 (64.3)	75 (76.5)		
Stage III	35 (35.7)	23 (3.5)	0.042	1.81 (0.97–3.38)
Mechanical ventilation (days)	13.7 ± 6.4	10.8 ± 5.3	0.005	0.17 (0.08–0.98)
Days to enteral feeds (median)	22.9 ± 12.3	23.2 ± 11.6	0.23	0.42 (0.28–1.62)
90-day parenteral nutrition dependence, *n* (%)	7 (7.1)	6 (6.1)	0.30	
NICU length of stay (days), mean ± SD	26.5 ± 13.2	23.2 ± 12.5	0.093	0.32 (0.18–1.09)
Bacterial sepsis, *n* (%)	18 (18.4)	12 (12.2)	0.16	
Albumin minimum (g/L, normal range, 35–50)	32.4 ± 6.2	33.7 ± 5.8	0.125	
Mortality, *n* (%)	28 (28.6)	20 (20.4)	0.10	1.56 (0.81–3.01)
Short bowel syndrome	7 (7.1)	3 (3.1)	0.17	

## Data Availability

The data used to support the findings of this study are available from the corresponding author upon request.
